# Vitamin D_3_ and gargling for the prevention of upper respiratory tract infections: a randomized controlled trial

**DOI:** 10.1186/1471-2334-14-273

**Published:** 2014-05-19

**Authors:** Emma C Goodall, Andrea C Granados, Kathy Luinstra, Eleanor Pullenayegum, Brenda L Coleman, Mark Loeb, Marek Smieja

**Affiliations:** 1 Department of Clinical Epidemiology and Biostatistics, McMaster University, Hamilton, Ontario, Canada; 2 Department of Medical Sciences, McMaster University, Hamilton, Ontario, Canada; 3 St. Joseph’s Healthcare, Hamilton, Ontario, Canada; 4 Child Health Evaluative Sciences, The Hospital for Sick Children, Toronto, Ontario, Canada; 5 Department of Microbiology, Mount Sinai Hospital, Toronto, Ontario, Canada; 6 School of Public Health, Faculty of Medicine, University of Toronto, Toronto, Ontario, Canada; 7 Department of Medicine, Institute for Infectious Disease Research, McMaster University, Hamilton, Ontario, Canada; 8 Department of Pathology & Molecular Medicine, McMaster University, Hamilton, Ontario, Canada; 9 Population Health Research Institute, McMaster University, Hamilton, Ontario, Canada

**Keywords:** Rhinovirus, Vitamin D3, Viral load, Gargling, Randomized controlled trial, Upper respiratory tract infection

## Abstract

**Background:**

We undertook a 2X2 factorial, randomized controlled trial (RCT) to assess whether vitamin D_3_ supplementation (10,000 international units per week) versus placebo and gargling versus no gargling could prevent viral, clinical upper respiratory tract infection (URTI) in university students.

**Methods:**

We randomized 600 students into 4 treatment arms: 1) vitamin D_3_ and gargling, 2) placebo and gargling, 3) vitamin D_3_ and no gargling, and 4) placebo and no gargling. Students completed weekly electronic surveys and submitted self-collected mid-turbinate nasal flocked swabs during September and October in 2010 or 2011. Symptomatic students also completed an electronic symptom diary. The primary and secondary outcomes were the occurrence of symptomatic clinical URTI and laboratory confirmed URTI respectively.

**Results:**

Of 600 participants, 471 (78.5%) completed all surveys while 43 (7.2%) completed none; 150 (25.0%) reported clinical URTI. Seventy participants (23.3%) randomized to vitamin D_3_ reported clinical URTI compared to 80 (26.7%) randomized to placebo (RR:0.79, CI_95_:0.61-1.03, p = 0.09). Eighty-five participants (28.3%) randomized to gargling reported clinical URTI compared to 65 participants (21.7%) randomized to the no gargling arm (RR:1.3, CI_95_:0.92-1.57, p = 0.19). Laboratory testing identified 70 infections (46.7 per 100 URTIs). Vitamin D_3_ treatment was associated with a significantly lower risk for laboratory confirmed URTI (RR: 0.54, CI_95_:0.34-0.84, p = 0.007) and with a significantly lower mean viral load measured as log_10_ viral copies/mL (mean difference: -0.89, CI_95:_ -1.7, -0.06, p = 0.04). Fewer students assigned to gargling experienced laboratory confirmed URTI, however this was not statistically significant (RR:0.82, CI_95_:0.53-1.26, p = 0.36).

**Conclusions:**

These results suggest that vitamin D_3_ is a promising intervention for the prevention of URTI. Vitamin D_3_ significantly reduced the risk of laboratory confirmed URTI and may reduce the risk of clinical infections.

**Trial registration:**

Clinical Trials Registration: NCT01158560.

## Background

Upper respiratory tract infection (URTI), which presents clinically as the common cold, is the most common human illness [1,2]. Multiple respiratory viruses are known to cause episodes of URTI, however rhinovirus has consistently been identified as the most common cause of the common cold [2,3]. While the majority of infections are mild and self-limiting, URTI can exacerbate existing medical conditions and can cause severe illness which may result in hospitalization or death [1,2,4].

Observational studies have consistently demonstrated an association between low vitamin D levels and greater frequency and severity of URTI in children and adults [5-11]. Results from several trials of vitamin D_3_ supplementation report reduced risk of infection, but only three studies have reported statistically significant findings [12-21]. The lack of consensus among these studies may reflect substantial heterogeneity across trials resulting from variation in participant populations, degree of vitamin D deficiency, the dose and duration of vitamin D supplementation, and definitions of URTI [12-21]. Additionally, several trials were limited by retrospective data collection and post-hoc analyses of trials with non-respiratory outcomes [12-14]. Consequently, there remains a need for rigorously-designed, large clinical trials to investigate the effect of vitamin D_3_ on URTI in a healthy population.

In Japan, daily gargling with water is recommended as a preventive measure against URTI. Results from a Japanese trial reported a 36% reduction in incident URTI amongst participants randomized to gargle with water three times daily compared to the control group [22].

The current study was designed to assess the effectiveness of vitamin D_3_ supplementation versus placebo, and of gargling versus no-gargling, for the prevention of URTI in university students.

## Methods

### Study design

We conducted a 2 × 2 factorial RCT of vitamin D_3_ versus placebo and gargling versus no-gargling with students at McMaster University, Hamilton, Ontario. Participants were enrolled during the first two weeks of September 2010 or 2011 and were followed to the end of October 2010 or 2011, respectively. Individuals were eligible for the study if they were enrolled at McMaster University, were ≥17 years, and lived with at least one student housemate. Participants with contra-indicated medical conditions (hypercalcemia, parathyroid disorder, chronic kidney disease, use of anticonvulsants, malabsorption syndromes, sarcoidosis), who were currently or planning to become pregnant, who were taking ≥1000 international units (IU)/day vitamin D_3_, or who were unable to swallow capsules were excluded. All participants provided written consent. The study protocol was approved by the Hamilton Health Sciences/Faculty of Health Sciences Research Ethics Board and was registered at clinicaltrials.gov (NCT01158560).

Participants completed a baseline questionnaire that collected demographic, health and lifestyle information and submitted a self-collected mid-turbinate flocked nasal swab (Copan Italia, Brescia Italy) [23]. Participants were then randomized to one of four allocation arms: 1) vitamin D_3_ and gargling, 2) vitamin D_3_ and no gargling, 3) placebo and gargling, or 4) placebo and no gargling. The study sample was stratified based on housing (in residence versus off-campus) and block randomization occurred within each stratum using a 1:1:1:1 allocation ratio. Only the study pharmacist knew the randomization scheme. The allocation was concealed using opaque, sealed, serially numbered envelopes. To prevent any violations of the allocation concealment, the envelopes could only be accessed by two study personnel who were not involved in their preparation. The envelopes were only accessed when both personnel were present, and the size of the randomization blocks was not known. The study was double-blind with respect to the vitamin D_3_/placebo intervention. Due to the nature of the gargling intervention, participants randomized to the gargling were not blinded. All other participants and study personnel remained blinded.

### Interventions

Participants were randomized to receive a container with eight capsules of either 10,000 IU of active vitamin D_3_ or identical placebo. All participants were instructed to take one pill weekly and received a weekly email reminder. Individuals randomized to the gargling intervention were asked to gargle with approximately 30 mL of tap water for 30 seconds twice daily. All participants received general lifestyle and health advice about the benefits of appropriate sleep, nutrition, hand hygiene, and exercise at the time of randomization. Intervention allocation occurred via serially numbered pill containers and opaque envelopes, containing gargling allocation, given directly to participants. The vitamin D was purchased from Euro-Pharm International Canada Inc. and the placebo contained only calcium carbonate. The pills were made aesthetically identical through use of a gelatin capsule.

### Assessments

Participants were asked to complete weekly online surveys which screened for URTI symptoms and to submit one self-collected nasal swab weekly. Participants with URTI were asked to complete a symptom survey for seven consecutive days following symptom onset and a follow-up survey 14 days after symptom onset. They were also asked to collect seven consecutive daily nasal swabs starting from symptom onset. Swabs were stored at room temperature in CyMol™ transport medium (Copan Italia, Brescia Italy), an alcohol-based medium which inactivates respiratory viruses on contact [24]. Only swabs submitted from symptomatic participants were tested for respiratory viruses. All other swabs were stored for separate studies, including investigations into asymptomatic illnesses.

### Outcome measures

The primary outcome was the incidence of clinical URTI, defined as the participant’s perception of a “cold” in conjunction with two or more symptoms (runny/stuffy nose, congestion, cough, sneezing, sore throat, muscle aches, or fever). Students were asked to immediately electronically report the onset of a “cold” and the weekly survey asked participants if they considered themselves to be sick. Adjudication by two clinicians was applied when participants reported symptoms but were uncertain if they were ill. These reports were deemed adjudicated events if 1) at least two symptoms were reported and included one of nasal congestion, sneezing, cough, sore throat, and wheezing, and 2) no additional information was provided that attributed the symptoms to another cause. Self-reported and adjudicated episodes of clinical URTI were considered ‘events’ if the onset occurred at least seven days after the participant’s randomization date.

Secondary outcomes included laboratory confirmed illness, viral load, and symptom duration and severity. Laboratory confirmed illness was determined by testing the Day 1 nasal swabs using an in-house enterovirus/rhinovirus polymerase chain reaction (PCR) and, if negative, a commercial multiplex PCR able to detect 16 respiratory viruses and viral subtypes (xTAG RVP FAST, Luminex, Austin TX). No testing was performed to identify bacterial agents. Viral load was determined for rhinovirus infections using quantitative PCR [25]. Symptom severity and duration were measured using the 21-item Wisconsin Upper Respiratory Symptom Survey [26]. Symptom severity was calculated as the sum of seven consecutive daily severity scores. Symptom duration was defined as the total number of days from symptom onset until the participant responded “I do not feel sick today”.

At enrolment, participants were asked about the frequency of hand washing before meals, average weekly hours of exercise, average hours of sleep per night, current vitamin supplement use and gargling habits, as well as asthma and smoking status to provide information for potential confounders, mediators, or moderators.

### Statistical analysis

We aimed to recruit 600 unique participants to ensure greater than 80% power to detect a 25% reduction (from 50% to 37.5%) in the proportion of students with URTI, with 10% over-recruitment to adjust for attrition. This study was powered to detect main effects and was underpowered to definitively investigate interactions.

Poisson regression with robust standard errors was used to assess our primary question of whether vitamin D_3_ or gargling could reduce the number of clinical URTIs experienced in those groups. This analysis was chosen in place of logistic regression since odds ratios overestimate treatment effects when incorrectly interpreted as risk ratios [27]. Robust standard errors were calculated in place of model based standard errors which are typically too large [27]. Multiple imputation, using the Markov chain Monte Carlo method, was conducted to address missing data. Information collected at baseline and through weekly surveys was used to predict missing values for independent and dependent variables [28]. The pooled imputed data was used to conduct an intention-to-treat analysis adjusted for randomization strata: housing, trial year, vitamin D_3_ and gargling allocation. Interaction between vitamin D_3_ use and gargling was investigated using a cross-product term. A complete case analysis, adjusted for the same variables, was performed as a sensitivity analysis. An identical complete-case analysis was conducted to assess the secondary outcome of laboratory confirmed infections. Symptom severity and viral load (log viral copies/mL) were compared by *t*-test. Cox regression was used to assess time to symptom resolution adjusted for the variables listed above. All analyses were planned *a priori*.

Results were considered statistically significant with *p* < 0.05. Statistical analyses were conducted using IBM SPSS statistical software version 20.0 (IBM SPSS Inc., Chicago, IL, USA).

## Results

### Participants

**Figure 1:**
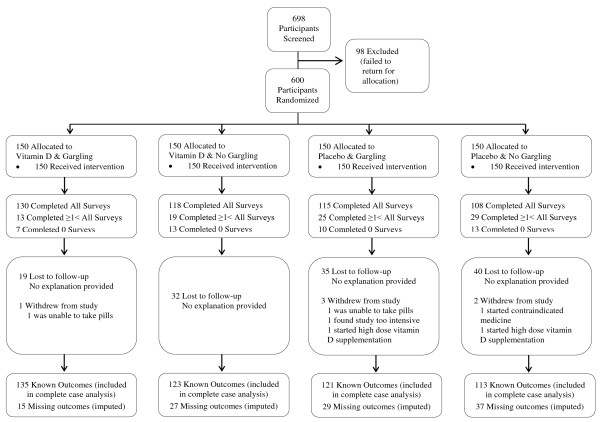
Flow diagram of participant randomization and follow-up during the McFLu2 COLD3 Prevention Trial, Hamilton, Canada; 2010 and 2011.

Six hundred ninety-eight prospective participants provided written consent. Of these, 600 completed the baseline survey, provided a nasal swab and were randomized into the study. Four hundred seventy one (78.5%) completed all weekly surveys, 86 (14.3%) completed at least one but not all, and 43 (7.2%) completed none (Figure 1). The median age of the participants was 19 years (interquartile range 18–20), 60% were first or second year undergraduate students, and 64% were female. Baseline characteristics were similar across the intervention arms (Table 1).

**Table 1:** Baseline characteristics of student participants across study arms, Hamilton, Canada, 2010 and 2011

	**Vitamin D & gargling(N = 150)**	**Vitamin D & No gargling (N = 150)**	**Placebo & gargling (N = 150)**	**Placebo & No gargling (N = 150)**
**Age, median years (IQR)**	**19 (18-20)**	**19 (18-21)**	**19 (18-20)**	**19 (18-21)**
**Female, No. (%)**	96 (64.0)	97 (64.7)	100 (66.7)	89 (59.3)
**Living in residence, No. (%)**	48 (32.0)	50 (33.3)	50 (33.3)	49 (32.7)
**Asthma, No. (%)**	14 (9.3)	16 (10.7)	16 (10.7)	12 (8.0)
**≥ 7 Hours sleep, No. (%)**	114 (76.0)	102 (68.0)	111 (74.0)	116 (77.3)
**Exercise, mean hours (SD)**	5.1 (4.2)	4.9 (3.5)	4.8 (4.9)	4.8 (4.0)
**Hand washing before meals, No.(%)**				
**Never**	34 (22.7)	30 (20.0)	36 (24.0)	42 (28.0)
**Usually**	75 (50.0)	71 (47.3)	68 (45.3)	70 (46.7)
**Always**	40 (26.7)	45 (30.0)	42 (28.0)	35 (23.3)
**Smoking, No. (%)**				
**Occasionally**	6 (4.0)	5 (3.3)	6 (4.0)	6 (4.0)
**Daily**	0	1 (0.7)	2 (1.3)	4 (2.7)
**Vitamin use, No. (%)**	30 (20.0)	40 (26.7)	34 (22.7)	21 (14.0)
**Daily gargling, No. (%)**	15 (10.0)	19 (12.7)	18 (12.0)	8 (5.3)

SD: Standard deviation, No: Number.

### Outcomes

#### Incidence of clinical and laboratory confirmed URTI

Clinical URTI and laboratory confirmation data was available for 492 (82%) and 489 (81.5%) participants, respectively. A total of 150 individuals experienced symptomatic (clinical) URTI with 106 episodes self-reported and 44 episodes established through adjudication. Laboratory testing identified viral pathogens in 70 infections, with rhinovirus being the predominant viral pathogen (61 cases), six cases of enterovirus and one case of coronavirus NL63. The distinction between rhinovirus and enterovirus was not established for two samples.

As shown in Table 2, fewer participants randomized to receive vitamin D_3_ (70 or 27%) reported a clinical URTI than those randomized to receive placebo (80 or 34%) (imputed analysis RR:0.80, CI_95_:0.63,1.02, p = 0.08; complete case analysis RR:0.79, CI_95_: 0.61,1.03, p = 0.09). Laboratory confirmed events were significantly lower in participants receiving vitamin D_3_ supplementation (26 or 10.2%) compared with those receiving placebo (44 or 18.9%) (RR:0.54, CI_95_:0.34,0.84, p = 0.007) (Table 2).

**Table 2:** Frequency, severity, duration and viral load (log viral copies/mL) associated with URTI according to vitamin D allocation

**Outcome measure**	**Clinical URTI**				**Laboratory confirmed URTI**			
	**Vitamin D (n = 258)**	**Placebo (n = 234)**	**Effect measure (95% CI)**	**P value**	**Vitamin D (n = 256)**	**Placebo (n = 233)**	**Effect measure (95% CI)**	**P value**
Imputed URTI episodes, No. (%)	91 (30.3)	114 (38.0)	^*^RR:0.80 (0.63-1.02)	0.08				
Complete case URTI episodes, No. (%)	70 (27.1)	80 (34.2)	^*^RR:0.79 (0.61-1.03)	0.09	26 (10.2)	44 (18.9)	^*^RR:0.54 (0.34-0.84)	0.007
^€^Viral load, mean (SD)					5.51 (1.6)	6.40 (1.2)		0.036
^†^Symptom duration, mean days, (SD)	6.0 (1.6)	6.2 (1.3)	^*^HR: 1.32 (0.59-2.90)	0.49	5.8 (1.5)	6.2 (1.2)	^*^HR: 1.30 (0.49-3.48)	0.59
^†^Symptom severity, mean (SD)	218.6 (124.0)	199.8 (108.1)		0.41	229.7 (110.3)	181.5 (91.9)		0.09

RR: relative risk, HR: Hazard ratio.

^*^The Poisson and Cox regression models were adjusted exclusively for randomization strata: vitamin D allocation, gargling allocation, year of participation and type of housing.

^€^Viral load was measured as log_10_ viral copies/mL.

^†^Symptom duration and severity was measured only in participants who self-reported illness (ie. not for adjudicated events), n = 106. For clinical URTI, n = 53 in each of the vitamin D and placebo groups. For laboratory confirmed URTI n = 22 and n = 34 in the vitamin D and placebo groups respectively.

Eighty-five (33.2%) participants assigned to gargle reported clinical URTI compared to 65 (27.5%) participants not assigned to gargle (imputed analysis RR: 1.1, CI_95_: 0.78,1.44, p = 0.69; complete case analysis RR: 1.3, CI_95_: 0.92,1.57, p = 0.19) (Table 3). Thirty-three (13.0%) participants assigned to gargle had a laboratory confirmed URTI compared to 37 (18.7%) participants randomized to the control arm; however this was not a statistically significant difference (Table 3) (RR: 0.82, CI_95_:0.53,1.26, p = 0.36).

**Table 3:** Frequency, severity, duration and viral load associated with URTI according to gargling allocation

**Outcome measure**	**Clinical URTI**				**Laboratory confirmed URTI**			
	**Gargling (n = 256)**	**Control (n = 236)**	**Effect measure (95% CI)**	**P value**	**Gargling (n = 254)**	**Control (n = 235)**	**Effect measure (95% CI)**	**P value**
Imputed URTI episodes, No. (%)	106 (35.3)	100 (33.3)	^*^RR: 1.1 (0.78-1.44)	0.69				
Complete case URTI episodes, No. (%)	85 (33.2)	65 (27.5)	^*^RR: 1.2 (0.92-1.57)	0.19	33 (13.0)	37 (18.7)	^*^RR:0.82 (0.53-1.26)	0.36
^€^Viral load, mean (SD)					6.24 (1.3)	5.95 (1.5)		0.43
^†^Symptom duration, mean days (SD)	6.2 (1.3)	6.0 (1.6)	^*^HR: 0.85 (0.38-1.89)	0.69	5.9 (1.3)	6.1 (1.4)	^*^HR: 0.43 (0.56-4.0)	0.43
^†^Symptom severity, mean (SD)	225.3 (123.9)	191.8 (105.1)		0.13	210.5 (105.3)	191.8 (98.8)		0.49

RR: relative risk, HR: Hazard ratio.

^*^The Poisson and Cox regression models were adjusted exclusively for randomization strata: vitamin D allocation, gargling allocation, year of participation and type of housing.

^€^Viral load was measured as log_10_ viral copies/mL.

^†^Symptom duration and severity was measured only in participants who self-reported illness (ie. not for adjudicated events), n = 106. For clinical URTI, n = 55 and n = 51 in the gargling and control groups respectively. For laboratory confirmed URTI n = 26 and n = 30 in the gargling and control groups respectively.

### Viral load

Viral load was established for 59 of 61 rhinovirus infections. The mean viral load in the vitamin D_3_ group was lower compared to those in the placebo group, with 5.51 log_10_ viral copies/mL versus 6.40 log_10_ viral copies/mL, respectively (mean difference: -0.89, CI_95:_ -1.7, -0.06, p = 0.04) (Table 2). The mean viral load in those assigned to the gargle group was 6.24 log_10_ viral copies/mL compared to 5.95 log_10_ viral copies/mL in the control group (mean difference: 0.37, CI_95_: -0.44, 1.02, p = 0.43) (Table 3).

### Symptom duration and severity of clinical and laboratory confirmed URTI

The mean duration of clinical and laboratory confirmed URTI was non-significantly lower in the vitamin D_3_ group compared to the placebo group (6.0 versus 6.2 days and 5.8 versus 6.2 days, respectively). Time to symptom resolution was not significantly different between the groups (clinical URTI HR: 1.3, CI_95_: 0.59, 2.90, p = 0.49; laboratory confirmed URTI HR: 1.3, CI_95_: 0.49,3.48, p = 0.59) (Table 2).

Gargling did not reduce the mean duration of symptoms or improve time to symptom resolution. Results were similar for clinical URTIs (HR: 0.85, CI_95_: 0.38,1.89, p = 0.69) and laboratory confirmed URTIs (HR:1.5, CI_95_:0.56,4.0, p = 0.43) (Table 3).

Mean symptom severity appeared to be greater in the vitamin D_3_ group for clinical and laboratory confirmed URTI (218.6 versus 199.8 and 229.7 versus 181.5, respectively) (Table 2). However, the difference was not statistically significant. Symptom severity also appeared to be greater in the gargling group for clinical and laboratory confirmed URTI, however this was not statistically significant (225.3 versus 191.8 and 210.5 versus 191.8, respectively) (Table 3).

## Discussion

Weekly supplementation with 10,000 IU of vitamin D_3_ in university students during September and October was associated with a non-significant, but potentially clinically important 20% risk reduction of clinical URTI. Importantly, vitamin D supplementation was associated with a statistically significant 46% risk reduction of laboratory confirmed URTI.

Results from previous trials of vitamin D_3_ supplementation for the prevention of URTI have been conflicting. Two placebo controlled RCTs in pediatric populations and one trial in adults with primary immune suppression have demonstrated that vitamin D_3_ significantly reduced the risk of clinical and lab-confirmed URTI and improved annual infection scores, respectively [17,18,20]. However, previous trials in healthy adult populations have not yielded statistically significant results. The effect estimates from our study are similar to those reported in pediatric trials. Urashima *et al.* reported that children, ages 6–15, receiving daily supplementation with 1200 IU vitamin D_3_ had a 42% lower risk of influenza A infection [17]. Camargo *et al.*, reported a 50% risk reduction in parent-reported URTI among children, ages 9–11, receiving daily supplementation with 300 IU of vitamin D_3_[18].

Consistent with previous studies in healthy adults, our primary analysis did not show that vitamin D_3_ significantly reduced the risk of clinical URTI; however the 20% relative risk reduction may be clinically relevant. Li-Ng *et al.* randomized 162 adults to 2000 IU vitamin D_3_ or placebo daily from December to March and reported no difference in self-reported URTI [15]. Laaksi and colleagues randomized 164 men to 400 IU vitamin D_3_ or placebo daily from October through March but did not detect a significant difference in the number of days absent from duty due to URTI [16]. However, the proportion of men who did not experience a URTI was significantly greater in the intervention group (51.3%) than in the control group (35.7%) [16]. Murdoch *et al*. followed 322 adults randomized to monthly doses of 100,000 IU vitamin D_3_ or placebo [19]. After 18 months, the study recorded 593 and 611 URTI episodes in the vitamin D_3_ and control groups respectively, however this was not a significant difference [19]. Rees *et al*. performed a substudy of 759 participants in a trial of colorectal adenoma chemoprevention. Participants were randomized to receive 1000 IU vitamin D3, calcium carbonate, both or placebo daily and were followed for an average of 13 months which covered two winter seasons. The authors reported that supplementation did not significantly reduce the incidence of URTI [21]. Bergman *et al*. followed 140 adults with increased susceptibility to respiratory infections at an immunodeficiency clinic. After one year of daily supplementation with either 4000 IU vitamin D_3_ or placebo, the authors reported a significantly lower infection score in the vitamin D_3_ group [20]. Notably, our *a priori* secondary outcome which assessed laboratory confirmed URTI demonstrated a significant 46% relative risk reduction associated with vitamin D_3_ supplementation. This result is consistent with two recent meta-analyses by Charan *et al.* and Bergman *et al.* which reported that vitamin D_3_ supplementation significantly decreased the odds of respiratory infections (pooled OR: 0.58, CI_95:_ 0.42-0.81, p = 0.001 and OR: 0.64, CI_95:_ 0.49-0.84, p = 0.0014, respectively) [29,30]. Although these results support the use of vitamin D_3_ supplementation, further meta-analysis, particularly an individual patient data meta-analysis, including the results from more recent trials and an exploration of heterogeneity between trials should be conducted.

Research has demonstrated that vitamin D plays a role in the innate immune response by stimulating the production of antimicrobial peptides, such as defensins and cathelicidins, and by enhancing the microbicidal action of monocytes and macrophages [31,32]. For this reason we hypothesized that vitamin D supplementation might reduce the amount of virus in infected persons. Our data support this hypothesis: mean log viral load was significantly lower in the vitamin D_3_ group compared to the placebo group.

Our study differed in several potentially important ways from previous studies. It is well established that vitamin D levels fluctuate seasonally [33]. Studies of Canadians have demonstrated a marked drop in vitamin D levels in the fall from their peak levels in the summer, and a high prevalence of low wintertime vitamin D levels [34,35]. To capture peak rates of rhinovirus infection in students, we initiated our intervention in early September, rather than during late autumn and winter months. Thus, serum vitamin D levels did not have the same opportunity to decline as they naturally would during the autumn. Rather than having to overcome depletion in vitamin D levels, our study may have maintained and potentially enhanced vitamin D levels. However, in the absence of blood tests this cannot be proven. Additional differences include the frequency and quantity of vitamin D_3_ supplementation. Our study used a relatively high dose of 10,000 IU of vitamin D_3_ per week ingested as a single dose, an average of approximately 1400 IU/day. Although the optimal dosing regimen remains uncertain, it has been acknowledged that adherence with daily supplements is often suboptimal and larger, less frequent doses may be an effective alternative [36,37]. A final methodological difference was our use of self-collected nasal swabs and laboratory confirmation of URTI. Our definition of clinical URTI may have been excessively broad and insufficiently specific which may have led to incorrectly classified events. It is possible that this definition captured episodes attributable to allergies or other causes, creating error in the statistical model and pushing the results towards a null effect.

Ultimately, our study was underpowered since the observed event rate was lower than predicted. This would contribute to larger variance in the estimates and uncertainty surrounding our results. This study was conducted over a relatively short period of time and while it captured peak rhinovirus activity, it was unable to capture URTI caused by other viruses. Consequently, it is uncertain whether vitamin D_3_ supplementation is beneficial for the prevention of non-rhinovirus URTI. An additional limitation was the lack of serum vitamin D testing without which we were unable to investigate whether individuals with lower baseline vitamin D levels benefitted incrementally compared to individuals with sufficient baseline vitamin D levels.

Gargling did not appear to reduce the risk of URTI in our study population, in contrast to previous reports [22]. Although our collection period captured peak rhinovirus activity, gargling may be more effective for pathogens which predominantly colonize the oropharynx. Additionally, we were unable to observe whether, or how often, gargling was practiced by participants; gargling may need to be carried out more frequently than twice daily to be beneficial.

## Conclusions

Our findings support the growing body of literature that proposes vitamin D_3_ as a promising intervention for the prevention of URTI in young adults. This study demonstrated that vitamin D_3_ can prevent laboratory confirmed acute, viral respiratory infections. While vitamin D supplementation may represent an effective, accessible and safe intervention for the prevention of URTI, many questions which might guide clinical practice remain unanswered. A rigorous systematic review and meta-analysis of current studies, particularly an individual patient data meta-analysis which would facilitate the evaluation of potential variable interactions, would be instrumental to the design of future trials in this field which may need to include a larger sample size, longer period of follow-up or different dosing regimens, and targeted populations who may benefit disproportionately from vitamin D supplementation.

## Abbreviations

CI95: 95% Confidence interval; IU: International units; HR: Hazard ratio; PCR: Polymerase chain reaction; RCT: Randomized controlled trial; RR: Relative risk; RRR: Relative risk reduction; URTI: Upper respiratory tract infection.

## Competing interests

The authors declare that they have no competing interests.

## Authors’ contributions

EG had full access to all study data and takes responsibility for the integrity of the data and the accuracy of the data analysis. EG, MS, EP and ML participated in the study design and development. Study conduct and supervision were conducted by EG and MS. EG, AG and KL were responsible for the acquisition of data. AG and KL conducted all laboratory testing. The analysis and interpretation of data was conducted by EG, EP, MS, ML and BC. EG drafted the manuscript and all authors critically reviewed the manuscript for intellectual content. All authors read and approved the final manuscript.

## Pre-publication history

The pre-publication history for this paper can be accessed here:

http://www.biomedcentral.com/1471-2334/14/273/prepub
